# Chromosome-scale diploid unphased assembly of horsemint (*Mentha longifolia*)

**DOI:** 10.1186/s12863-025-01375-6

**Published:** 2025-10-31

**Authors:** Meric C. Lieberman, Luca Comai, Isabelle M. Henry

**Affiliations:** Department of Plant Biology and Genome Center, University of California, 451 Health Science Dr, Davis, CA 95616 USA

**Keywords:** Genome sequencing, Mint, *Mentha longifolia*, Chromosome, Oil, Genome assembly

## Abstract

**Objectives:**

Mint oils are essential oils with many commercial applications. Mint oils are harvested from peppermint or spearmint plants. Spearmint (*Mentha spicata*) is an allotetraploid, hybrid between diploid parents *Mentha suaveolens* (apple mint) and *Mentha longifolia* (horse mint). Peppermint comes from a second hybridization event between spearmint and octoploid *Mentha aquatica* (water mint). Here we present a chromosome-scale diploid unphased assembly of a clone of a *Mentha longifolia*. Combined with the previously assembled *M. suaveolens* and a previous consensus assembly of a genetically more distant clone of *M. longifolia*, these assemblies provide valuable tools for trait mapping and understanding the genomic composition of commercial hybrid genomes and their relative contribution to traits important to the mint industry.

**Data description:**

A two haplotypes Hifiasm assembly of the genome of *M. longifolia* was generated from 105 x consensus coverage of PacBio long reads. The hifiasm assembly included 346 contigs and exhibited an N50 of 30.5 Mb. Two sets of pseudochromosomes were constructed by comparison to the previously published genome of *Mentha suaveolens*, resulting in 20 superscaffolds and 8 partial scaffolds, for an overall genome size of 734 Mb. Illumina RNA-Seq libraries from a mixed set of *Mentha* species were used to annotate the genome.

## Objectives

Spearmint and peppermint produce essential oils in specialized structures present on the surface of their leaves called glandular trichomes [1]. Mint oils, harvested by distillation, are used in a wide range of commercial products as the composition and resulting flavor of these oils vary tremendously depending on the mint species or even clone [2]. The two mint species most commonly grown commercially are spearmint and peppermint. Spearmints are known for producing an oil with a sweeter flavor, associated with the presence of (-)-carvone, the most abundant compound in spearmint oil. Peppermints produce an oil with a cooling effect associated with the presence of (-)-menthol. The exact composition of the oil produced by a specific mint clone is very difficult to predict, partially because of the complex genetic make-up of spearmint and peppermint, both interspecific polyploids [3]. Providing genomic information from the parental species that contributed to spearmint and peppermint is a first step in building tools for understanding and potentially manipulating the mint oil pathways.

Spearmint and peppermint are allopolyploids, with contributions from two and three different parental genomes, respectively [4]. Specifically, spearmints are allotetraploids originating from a cross between two diploid species called apple mint (*M. longifolia*) and horsemint (*M. suaveolens*). Peppermints come from an additional cross between spearmint and auto-octoploid water mint (*M. aquatica*). A chromosome-scale reference sequence for a clone of *M. suaveolens* was recently published [5], as well as a haplotype-resolved assembly of a different *M. suaveolens* clone [6]. A chromosomes-scale reference genome was developed recently for a *M. longifolia* clone as well [7] but phylogenetic analysis of a panel of clones of *M. suaveolens* and *M. longifolia* revealed that the sequenced *M. longifolia* clone was distantly related from all others analyzed [8] and might not constitute a good representative parental genome for spearmint or peppermint. Therefore, we elected to develop a reference sequence for another *M. longifolia* clone, more closely related to the other parental clones. The clone selected, PI 557,755, is characterized as resistant to Verticillium wilt (score of 0.3), and produces an oil that is rich in trans-piperitone oxide (43.3%), cis-piperitone oxide (19.7%) and 1,8-cineole (7.0%) [9].

## Data description

A total of 3.2 million PacBio HiFi reads (average length = 16.4 kb) were acquired and assembled using hifiasm, with *purge duplicate level* set to zero [10]. This resulted in a 758 Mb assembly, composed of 346 contigs and with an N50 of 30.5 Mb. To scaffold the contigs into pseudochromosomes, the largest 29 scaffolds (size > 4 Mb) were mapped using minimap2 [10] to the chromosome-level assembly of *M. suaveolens* [5]. All contigs uniquely mapped to a specific chromosome. In one case, two contigs were joined into a single larger chromosome scaffold based on the minimap2 results. Of the 12 chromosomes in the mint genome, we obtained two large chromosome haplotype contigs for 10 of the chromosomes, which we randomly named haplotype A or B for each chromosome. The remaining 2 chromosomes were each assigned two near full-length contigs (randomly named haplotype A or B) and two smaller contigs (randomly named haplotypes C or D). Based on the minimap2 results, in each case, one large and one small contig are expected to create a full-length chromosome contig, suggesting that our assembly does contain contigs that cover two full-length haplotypes in all cases. For those two chromosome types, we could not determine how to phase the contigs together so we did not merge them and chose to retain those four contigs separately in the final assembly. They are thus assigned to their respective chromosome types but not assigned to a specific haplotype. The resulting genomic reference contained 28 superscaffolds with an N50 of 30.5 Mb, and a total size of 734 Mb.

This genomic assembly was analyzed using the BUSCO eudicots_odb10 dataset, which reported the following statistics: 97.5% complete, 0.3% fragmented, and 3.3% missing BUSCO(s) [11]. This genomic assembly was next processed by the braker3 pipeline [12] using reads from 49 RNA-Seq libraries, totaling 1.9 billion 150 PE reads. These libraries correspond to a variety of tissue types sampled from *Mentha longifolia*, *Mentha suaveolens*, *Mentha aquatica*, *Mentha piperita*, and *Mentha spicata* [13]. Depending on the clone, this included tissue from stems, runners, buds, flowers, developing leaves, mature leaves and roots, as well as mature leaves sampled from field grown plants. The RNA-Seq libraries were combined and the reads mapped to the draft genomic reference using hisat2 [14]. The resulting file was converted into a bam file using samtools [15] and used as input for braker3. Braker3 generated a CDS annotation containing 205,350 transcripts, representing a coding space of 220 Mb. These CDS transcripts were then input into BioBam’s Omicsbox (Biobam Bioinformatics) for functional annotation. This included mapping to NCBI’s NR database, InterProScan protein mapping, and GO Annotation. The resulting annotation included 146,686 transcripts with at least one type of functional annotation, and 58,664 with no associated annotation data. These transcripts were further subdivided into two randomly assigned haplotypes containing 75,359 and 71,327 transcripts each. The average BUSCO scores for these two transcript sets were 94.43% complete, 1.48% fragmented, and 4.08% missing [11] The genomic reference was also annotated for transposable elements using EDTA [16] (Table 1).

**Table 1:** Overview of data files/data sets

Label	Name of data file/data set	File types (file extension)	Data repository and identifier (DOI or accession number)
*Data file 1*	Assembled genome	*Fasta file (.fa)*	Figshare: 10.6084/m9.figshare.28457522 [17]
*Data file 2*	Predicted genes	*Gff3 file (.gff3)*	Figshare: 10.6084/m9.figshare.28457522 [17]
*Data file 3*	Predicted genes – CDS	*Fasta file (.fa)*	Figshare: 10.6084/m9.figshare.28457522 [17]
*Data file 4*	Predicted Functional CDS, one haplotype	*Fasta file (.fa)*	Figshare: 10.6084/m9.figshare.28457522 [17]
*Data file 5*	Predicted Functional CDS, second haplotype	*Fasta file (.fa)*	Figshare: 10.6084/m9.figshare.28457522 [17]
*Data file 6*	Predicted repetitive sequences	*Gff3 file (.gff3)*	Figshare: 10.6084/m9.figshare.28457522 [17]
*Data file 7*	Assembled genome	*Fasta file (.fa)*	NCBI GenBank Assembly GCA_052575305.1 https://identifiers.org/ncbi/insdc.gca:GCA_052575305.1 [18]
*Data file 8*	Assembled genome	*Fasta file (.fa)*	NCBI GenBank Assembly GCA_05257335.1 https://identifiers.org/ncbi/insdc.gca:GCA_052575335.1 [19]
*Data set 1*	*Raw RNA-seq reads*	*Fastq file (.fq)*	NCBI BioProject Accession number SRP565976 https://identifiers.org/insdc.sra:SRP565976 [20]
*Data set 2*	*Raw PacBio reads*	*PacBio basecall format (fq)*	NCBI BioProject Accession number SRP565976 https://identifiers.org/insdc.sra:SRP565976 [20]

## Limitations

The annotation was based on 49 libraries representing 9 tissue types. It is possible that some genes were not detected and annotated because they are not significantly expressed in the tissue types and condition sampled.

The annotation was based on transcriptome libraries from other mint clones, not the one sequenced. It is possible that specific genes present only in the sequenced clone will be missing from the annotation.

Additionally, many of the annotated transcripts did not generate any functional or GO annotation links, suggesting that they might have been incorrectly annotated or that they are novel. The quality of the mapped annotations therefore ranged from no transcript functional information up to transcripts with protein blast hits, GO annotation, and protein functional domain(s).

Finally, our study does not include any long-range interaction sequence data (Hi-C or other). Therefore, it is likely that haplotype-switches are present in the assembled chromosomes.

## Data Availability

The data described in this Data note can be freely and openly accessed at the National Center for Biotechnology Information Short Read Archive and FigShare as described in Table 1. The available datasets are summarized in Table 1 (https://bmcgenomdata.biomedcentral.com/articles/10.1186/s12863-023-01181-y) [1]. The *Mentha longifolia* clone used in the study, *Mentha longifolia (L.) Huds. subsp. capensis (Thunb.) Briq.* (Pl 557755, CMEN17), can be obtained from the USDA Mint Germplasm Collection (Corvallis, OR, USA).

## Abbreviations

HiFi: High-fidelity
BUSCO: Benchmarking Universal Single-Copy Orthologs
